# Reactive Oxygen Species as Key Molecules in the Pathogenesis of Alcoholic Fatty Liver Disease and Nonalcoholic Fatty Liver Disease: Future Perspectives

**DOI:** 10.3390/cimb47060464

**Published:** 2025-06-17

**Authors:** Zhiqing Zhang, Hong Yang, Fei Han, Peng Guo

**Affiliations:** 1 Department of Hepatobiliary Surgery, The Third Affiliated Hospital, Chongqing Medical University, Yubei, Chongqing 401120, China; cqmuzzq@163.com (Z.Z.); yunghhong@163.com (H.Y.); 2 School of Public Health, Chongqing Medical University, Chongqing 400016, China

**Keywords:** reactive oxygen species, alcoholic fatty liver disease, nonalcoholic fatty liver disease

## Abstract

Reactive oxygen species (ROS) are central to the progression of alcoholic fatty liver disease (ALD) and nonalcoholic fatty liver disease (NAFLD). In ALD, ROS arise from alcohol metabolism (CYP2E1 and ADH/ALDH2), causing oxidative damage and fibrosis. In NAFLD, mitochondrial dysfunction, ER stress, and lipotoxicity drive ROS overproduction due to metabolic dysregulation. Both diseases share ROS-mediated pathways, including mitochondrial/ER dysfunction, inflammation, and impaired lipid metabolism, accelerating steatosis to cirrhosis and cancer. Antioxidants, ER modulators, and lifestyle changes show therapeutic potential but require further clinical validation. Future research should leverage multi-omics and targeted therapies to optimize ROS-focused interventions for ALD and NAFLD.

## 1. Introduction

In recent years, significant global increases in the incidence of both alcoholic fatty liver disease (ALD) and nonalcoholic fatty liver disease (NAFLD) have been witnessed, establishing them as leading causes of non-viral liver disease. ALD shows a strong correlation with chronic excessive alcohol consumption, particularly prevalent among heavy drinkers in Western male populations [1]. In parallel, NAFLD has emerged as the hepatic component of metabolic syndrome, closely associated with obesity and type 2 diabetes. With approximately 25% of adults worldwide affected, the prevalence of NAFLD continues to rise in tandem with growing obesity and diabetes rates. Collectively, ALD and NAFLD now surpass viral hepatitis as the most burdensome liver diseases globally [2].

The pathogenesis of ALD involves reactive oxygen species (ROS) generated through multiple pathways of alcohol metabolism. These ROS, along with alcohol-derived metabolites, inflict direct hepatocellular damage while promoting hepatic inflammation and fibrosis [3]. Similarly, NAFLD progression is driven by oxidative stress stemming from insulin resistance and dysregulated lipid metabolism, both hallmark features of the disease [4]. ROS not only cause hepatocyte injury but also initiate and amplify inflammatory signaling pathways, creating a self-perpetuating cycle of oxidative damage and chronic inflammation.

Clinically significant is the increasing population with concurrent NAFLD and alcohol consumption. These patients demonstrate accelerated progression to liver fibrosis, cirrhosis, and hepatocellular carcinoma. Current evidence implicates ROS as a critical mediator in the exacerbated disease progression observed when NAFLD coexists with alcohol use. Therefore, elucidating ROS mechanisms in these overlap syndromes is crucial for understanding their rapid progression and may yield novel therapeutic approaches. This review examines ROS involvement in both ALD and NAFLD progression, compares their sources and mechanistic roles, evaluates antioxidant-based therapies, and explores future research directions targeting ROS in these diseases.

## 2. ROS Pathogenesis in ALD and NAFLD

ROS represent a class of oxygen-derived free radicals—including superoxide (O₂^−^), hydrogen peroxide (H_2_O_2_), hydroxyl radical (OH), and singlet oxygen (^1^O_2_)—that play dual roles in liver physiology and pathology. These reactive molecules originate primarily from the following three key sources: (1) mitochondrial electron leakage during oxidative phosphorylation; (2) NOX enzyme activity; and (3) cytochrome P450 metabolism (particularly CYP2E1). While serving as crucial second messengers in normal cell signaling and host defense, ROS become pathogenic when their production overwhelms cellular antioxidant capacity [5].

The liver employs specialized cellular systems for ROS generation and regulation: hepatocytes maintain redox balance through tightly controlled mitochondrial ROS production coupled with efficient scavenging systems; Kupffer cells utilize NOX-derived ROS as defensive weapons during immune activation; and activated hepatic stellate cells (HSCs) leverage ROS signaling to drive fibrotic responses during tissue repair [6].

The pathophysiological impact of specific ROS follows distinct mechanisms: signaling ROS (O_2_^−^/H_2_O_2_): modulate inflammatory cascades at physiological levels; destructive ROS (OH): cause indiscriminate macromolecular damage through membrane lipid peroxidation and DNA strand breaks; and dual-role H_2_O_2_: functions as a redox regulator at low concentrations but triggers oxidative injury when accumulated [6].

Additionally, since nitric oxide (NO) acts as an intercellular messenger with high diffusibility in the body, its reaction with O₂⁻ forms a unique type of ROS—peroxynitrite (ONOO−), which also belongs to reactive nitrogen species (RNS). ONOO− can attack all biomolecules, causing not only oxidative damage but also nitrative damage [7]. Among the most destructive oxidant species in cells are ·OH and ONOO− [8].

### 2.1. ROS Production in NAFLD (Figure 1)

NAFLD represents a dynamic disease continuum, progressing from simple hepatic steatosis to the more aggressive inflammatory condition of nonalcoholic steatohepatitis (NASH). This progression carries significant clinical implications, as NASH-related cirrhosis demonstrates markedly higher HCC incidence compared to other chronic liver diseases [9].

The liver serves as the metabolic command center for lipid homeostasis, uniquely coordinating fatty acid uptake, de novo lipogenesis, β-oxidation, and very-low-density lipoproteins (VLDLs)-mediated lipid export. Hepatocytes maintain lipid balance through three primary pathways: (1) mitochondrial β-oxidation for energy production, (2) VLDL assembly and secretion for peripheral distribution, and (3) lipid droplet storage for temporary buffering [10].

The following two pathophysiological drivers dominate NAFLD development:

(1) Metabolic dysregulation: Approximately 15% of hepatic fatty acids are derived directly from dietary intake. Chronic overnutrition leads to excessive lipid accumulation in hepatocytes, forming lipid droplets. This stored fat can subsequently be broken down into free fatty acids (FFAs), further contributing to hepatic stress and inflammation [11].
(2) Insulin resistance: The accumulation of lipid droplets and FFAs from metabolic dysregulation can impair insulin signaling. This weakened insulin response reduces the suppression of adipocyte lipolysis, leading to an excessive release of FFAs into the bloodstream. These FFAs are then transported to the liver, compounding the cycle of fat accumulation and liver dysfunction [12,13].

**Figure 1:**
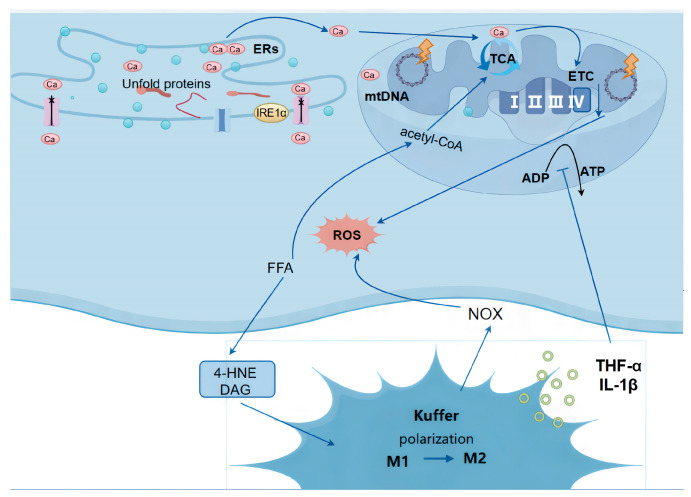
Mechanisms of ROS generation in NAFLD.

This metabolic perfect storm triggers multiple hepatotoxic mechanisms: mitochondrial dysfunction from FFA overload, ER stress response activation, and lipotoxicity-mediated cellular damage [14]. Together, these processes create a self-perpetuating cycle of oxidative stress through three well-characterized ROS-generating pathways (Figure 1):

#### 2.1.1. Mitochondrial Dysfunction

Hepatocyte mitochondria serve two critical metabolic functions: ATP production and fatty acid β-oxidation. Under normal physiological conditions, mitochondrial respiration generates ROS as natural byproducts. The electron transport chain (ETC), composed of enzyme complexes and mobile electron carriers (coenzyme Q and cytochrome c), typically leaks only 2–3% of electrons during aerobic respiration, resulting in physiological ROS levels [15].

In NAFLD pathogenesis, lipid metabolism dysregulation leads to excessive intracellular fatty acid accumulation, which significantly impairs mitochondrial function. When the ETC reaches saturation from FFAs β-oxidation, electron leakage increases dramatically, generating pathological ROS levels [16]. This oxidative stress is further exacerbated through multiple mechanisms: FFA overload compromises mitochondrial membrane integrity, while inflammatory cytokines from activated Kupffer cells and HSCs in NASH cause mtDNA damage, impairing mitochondrial protein synthesis and ETC complex function. FFAs induce ER stress that disrupts cellular Ca^2+^ homeostasis, promoting mitochondrial permeability transition pore (mPTP) opening. The subsequent Ca^2+^ influx into mitochondria hyperactivates the respiratory chain, tricarboxylic acid cycle (TCA), and oxidative phosphorylation, creating an “electron traffic jam” that increases ROS production [17,18].

#### 2.1.2. Endoplasmic Reticulum Stress (ER Stress)

Endoplasmic reticulum stress (ERS) is a pathological process triggered by the abnormal accumulation of misfolded or unfolded proteins within the ER lumen. Its core inducers include oxidative stress, metabolic disturbances, and toxic insults [19,20]. While ERS initially functions as an adaptive or protective cellular mechanism, persistent or excessive protein misfolding leads to cellular damage and stress. In NAFLD, ERS is primarily driven by lipotoxicity: a high-fat diet or obesity causes excessive accumulation of free fatty acids (FFAs) and cholesterol in hepatocytes. Concurrently, during NASH progression, inflammatory cytokines disrupt ER proteostasis, cross-activating ER stress pathways. When combined with lipid metabolism dysfunction, ethanol-induced suppression of lipophagy further exacerbates FFA accumulation, synergistically promoting ERs.

The unfolded protein response (UPR) constitutes the core mechanism of ERS, mediated by three ER transmembrane sensors: protein kinase R-like endoplasmic reticulum kinase (PERK), inositol-requiring enzyme 1α (IRE1α), and activating transcription factor 6 (ATF6) [21]. Calcium (Ca^2+^) dysregulation is a key contributor to ERS-induced ROS generation. UPR activation stimulates IRE1α, leading to Ca^2+^ leakage from the ER. Additionally, sarco/endoplasmic reticulum Ca^2+^-ATPase (SERCA), responsible for pumping cytosolic Ca^2+^ back into the ER, becomes impaired during ERS. This results in ER Ca^2+^ depletion and cytosolic/mitochondrial Ca^2+^ overload, further driving ROS production [22].

Moreover, ERs suppresses the antioxidant defense system: while PERK activation transiently induces Nrf2 expression, chronic ERs promotes Nrf2 degradation, reducing antioxidant enzymes such as SOD and catalase (CAT) [23]. Additionally, IRE1α upregulates NADPH oxidase 4 (NOX4), further amplifying ROS generation [18].

#### 2.1.3. Lipotoxicity

Lipotoxicity results from excessive accumulation of FFAs and their metabolites (ceramides, diacylglycerols (DAG), and 4-hydroxynonenal(4-HNE)) in hepatocytes, causing direct cellular damage and metabolic dysfunction. This process drives Kupffer cell polarization from anti-inflammatory (M2) to pro-inflammatory (M1) phenotypes, which then generate excessive ROS via NADPH oxidase (NOX) activation while secreting inflammatory cytokines (TNF-α and IL-1β) that exacerbate oxidative stress. These mediators concurrently disrupt mitochondrial membrane potential and impair ATP production, creating a self-amplifying cycle of metabolic dysfunction and ROS overproduction [24,25].

Three core mechanisms of hepatocyte injury in NAFLD: mitochondrial dysfunction (excessive FFAs cause electron leakage in the ETC and ROS burst), endoplasmic reticulum stress (ERS triggers Ca^2+^ dysregulation and NOX4 upregulation via the UPR pathway), and lipotoxicity (FFA metabolites activate Kupffer cell M1 polarization, exacerbating oxidative stress through NOX). Key molecules labeled in the figure—including the TCA cycle, ATP, ROS, and the M1 → M2 polarization process—collectively illustrate the vicious cycle of oxidative stress and metabolic dysregulation in NAFLD.

### 2.2. ROS Production in ALD (Figure 2)

ALD is caused by chronic excessive alcohol consumption and is a major cause of chronic liver disease worldwide [26]. While some patients may develop severe ALD, including alcoholic steatohepatitis (ASH), cirrhosis, and HCC, the pathogenesis of ALD remains unclear despite significant progress over the past two decades, and there are currently no FDA-approved drugs for ALD treatment.

The development of ALD is rooted in the disruption of hepatic metabolic homeostasis following alcohol absorption in the intestine and subsequent metabolism in the liver and other organs. Hepatic steatosis occurs in over 90% of heavy drinkers and is characterized by lipid accumulation in hepatocytes [27]. Multiple mechanisms contribute to hepatic steatosis, including impaired mitochondrial fatty acid β-oxidation, lipid migration from extrahepatic tissues to the liver, and alterations in lipid metabolism-related transcription factors. ASH is defined by specific histological features, including marked steatosis, inflammatory cell infiltration, pericellular fibrosis, and hepatocyte ballooning degeneration. Among ASH patients, 8–20% progress to cirrhosis, and 3–10% of those with alcohol-related cirrhosis develop HCC [26].

**Figure 2:**
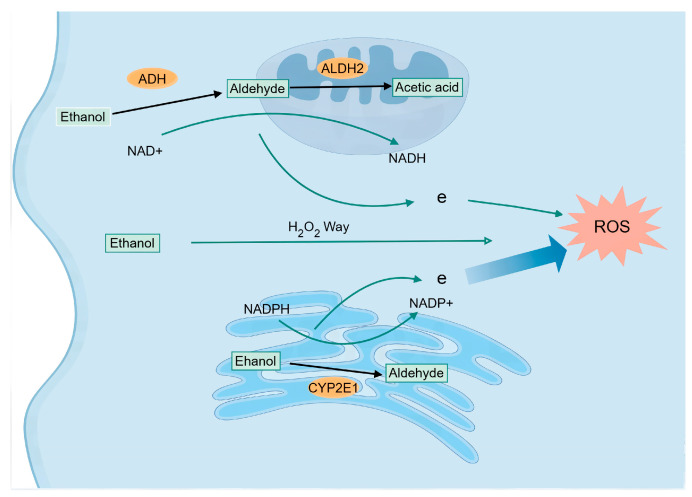
Mechanism of ROS generation in ALD.

ALD shares some similarities with NAFLD in terms of pathogenesis, but since the liver is the primary site of alcohol metabolism, the main source of ROS in ALD is derived from alcohol metabolism [28]. There are three major pathways through which alcohol metabolism generates ROS in the liver (Figure 2).

#### 2.2.1. Alcohol Dehydrogenase (ADH) and Aldehyde Dehydrogenase 2 (ALDH2) Pathway

Ethanol is first metabolized to acetaldehyde by ADH, which is then further converted to acetate by ALDH2. Both steps generate reduced nicotinamide adenine dinucleotide (NADH), leading to an increased NADH/NAD^+^ ratio. This imbalance inhibits the mitochondrial respiratory chain, resulting in enhanced electron leakage during electron transfer and subsequent ROS production.

#### 2.2.2. Microsomal Ethanol Oxidizing System (MEOS)

MEOS represents an alternative pathway for alcohol metabolism, primarily occurring in the endoplasmic reticulum. Cytochrome P450 2E1 (CYP2E1), the key enzyme in MEOS, catalyzes the oxidation of ethanol to acetaldehyde. During this process, CYP2E1 requires NADPH as an electron donor, which is oxidized to NADP^+^ while generating superoxide anion (O_2_^−^). These superoxide anions are further converted to hydrogen peroxide (H_2_O_2_) and other ROS. This pathway constitutes the most significant source of ROS in alcohol metabolism.

#### 2.2.3. Catalase Pathway

The catalase pathway primarily operates during chronic alcohol consumption and metabolizes ethanol by decomposing hydrogen peroxide. This pathway generates relatively lower levels of ROS compared to the other two mechanisms.

Three major pathways of ethanol metabolism generate ROS in ALD. The first is the ADH- and ALDH2-mediated metabolic pathway, where ethanol is converted to acetaldehyde and then to acetate, simultaneously producing NADH that leads to redox imbalance. The second is the MEOS, catalyzed by the CYP2E1 enzyme, which directly generates ROS intermediates. The third is the catalase pathway, producing relatively lower levels of ROS. Key molecules labeled in the figure include metabolic enzymes, cofactors, and ROS, demonstrating the molecular correlation between alcohol metabolism and oxidative stress.

### 2.3. Similarities and Differences in ROS Production Between ALD and NAFLD

#### 2.3.1. Common Sources of ROS in ALD and NAFLD

Both ALD and NAFLD involve excessive ROS production due to increased electron leakage in the mitochondrial ETC, driven by alcohol metabolism in ALD and FFA metabolism in NAFLD. Additionally, both diseases can induce endoplasmic reticulum (ER) stress, leading to Ca^2+^ dyshomeostasis and subsequent mitochondrial ROS generation. When progressing to ASH or NASH, both conditions activate Kupffer cells and promote the release of pro-inflammatory cytokines (e.g., TNF-α and IL-6), further enhancing ROS production.

#### 2.3.2. Key Differences in ROS Generation Between ALD and NAFLD

(1) Distinct Initial Triggers of ROS Production

ALD: Alcohol metabolism is the primary source of ROS, particularly through the ADH/ALDH2 and CYP2E1 pathways, which directly generate large amounts of ROS.

NAFLD: Insulin resistance and dysregulated lipid metabolism are the main drivers of ROS production. FFA overload leads to mitochondrial dysfunction and ER stress, indirectly increasing ROS levels.

(2) Different Dominant Pathways of ROS Generation

In ALD:

ADH/ALDH2 Pathway: Alcohol metabolism generates NADH, disrupting the NADH/NAD^+^ ratio, inhibiting the mitochondrial respiratory chain, and increasing electron leakage.

CYP2E1 Pathway: CYP2E1-mediated alcohol metabolism produces substantial superoxide anion (O₂⁻) and hydrogen peroxide (H_2_O_2_), representing the dominant ROS-generating mechanism in ALD.

In NAFLD:

Mitochondrial β-Oxidation: FFA overload accelerates mitochondrial β-oxidation, overloading the ETC and increasing electron leakage.

ER Stress: FFAs induce ER stress, causing Ca^2+^ leakage and subsequent mitochondrial ROS production.

Kupffer Cells Activation: FFAs and lipid metabolites directly damage hepatocytes and activate Kupffer cells to produce ROS.

See Table 1 for a comparative summary.

## 3. Mechanisms of ROS Action in ALD and NAFLD

### 3.1. Shared Mechanisms of ROS in ALD and NAFLD Pathogenesis (Figure 3)

**Figure 3:**
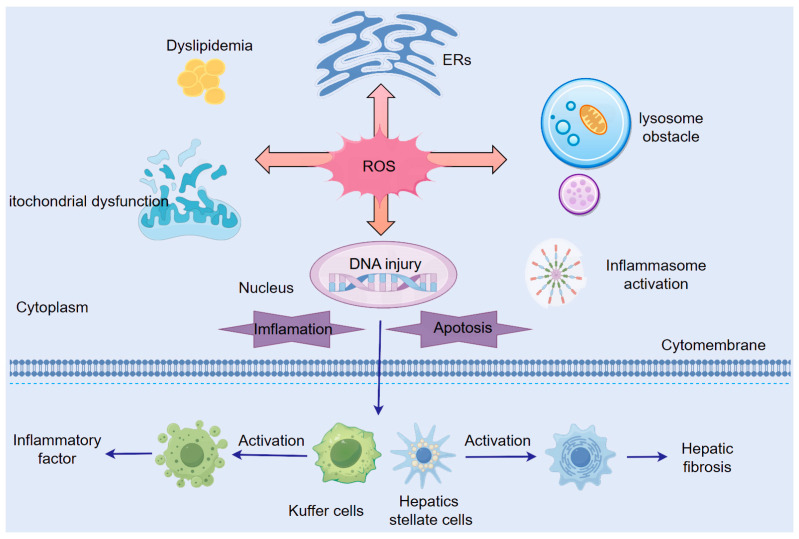
Common mechanisms of ROS in ALD and NAFLD.

Despite their distinct etiologies, ALD and NAFLD converge through ROS-mediated mechanisms, where oxidative stress damages mitochondria, ER, and lysosomes while simultaneously activating inflammatory pathways and exacerbating lipid metabolic dysfunction, collectively driving disease progression from steatosis to hepatitis, fibrosis, and ultimately hepatocellular carcinoma (Figure 3).

#### 3.1.1. ROS-Induced Mitochondrial Dysfunction

Mitochondria serve as both the primary source and key target of intracellular ROS. Excessive ROS induce mitochondrial membrane lipid peroxidation, disrupting the ETC and impairing oxidative phosphorylation. This oxidative stress reduces levels of PTEN-induced putative kinase 1 (PINK1), a protective mitochondrial serine/threonine–protein kinase, while concurrently causing abnormal mitochondrial morphology, diminished membrane potential, and decreased activities of Complex I and IV—collectively exacerbating ETC dysfunction and electron leakage to generate more ROS [15].

The vulnerability of mtDNA—lacking histone protection and having limited repair capacity—makes it particularly susceptible to ROS-mediated damage. ROS (e.g., O_2_^−^ and ·OH) directly attack mtDNA, inducing base modifications, strand breaks, and cross-linking, which compromise mtDNA integrity and disrupt mitochondrial protein synthesis. Furthermore, ROS-generated lipid peroxidation products (e.g., 4-HNE) bind to and inactivate mtDNA, while ROS-induced loss of mitochondrial membrane potential promotes Ca^2+^ influx, aggravating mtDNA damage [29].

ROS also trigger hepatocyte apoptosis through multiple pathways. Oxidative modification of anti-apoptotic Bcl-2 family proteins (e.g., Bcl-2 and Bcl-xL), inhibiting their function and promoting BAX/BAK activation. p53-mediated upregulation of BAX expression, driving BAX/BAK oligomerization to form mitochondrial outer membrane pores, increasing permeability and cytochrome C release—a pivotal step in activating the apoptosome and caspase cascade [30,31].

Additionally, ROS-induced mitochondrial damage and lipid peroxidation activate ferroptosis pathways, further accelerating hepatocyte death [32].

#### 3.1.2. ROS-Induced Endoplasmic Reticulum Stress (ER Stress)

ROS promote ERs by oxidizing amino acid residues (e.g., cysteine, methionine, and histidine) in proteins, leading to structural and functional ER damage. Cysteine residues (-SH) are particularly susceptible to ROS attack, where oxidation by H_2_O_2_ or OH generates sulfenic (-SOH), sulfinic (-SO_2_H), or sulfonic (-SO_3_H) acid modifications. These oxidative changes disrupt disulfide bonds (-S-S-), inducing protein misfolding, aggregation, or degradation, thereby exacerbating the UPR. This establishes a vicious cycle of ERS amplification, Ca^2+^ dyshomeostasis, and mitochondrial ROS release [33].

ERS Triggers Pro-Inflammatory Cascades

The three major UPR sensors—PERK, IRE1α, and ATF6—drive pro-inflammatory cytokine release through multiple pathways [34]:

(1) IRE1α upregulates TNF-α and IL-6 via its downstream target, spliced X-box binding protein 1 (XBP1s) [35].
(2) ER stress activates the IκB kinase (IKK) complex, inducing NF-κB nuclear translocation and subsequent TNF-α/IL-6 production [36].
(3) IRE1α recruits TRAF2, activating the JNK pathway to amplify cytokine release [37].

This sustained inflammation promotes HSC activation and fibrotic collagen deposition, accelerating NASH progression and liver fibrosis [19].

ERS Drives Metabolic Dysregulation

UPR intersects with cytosolic stress kinases (JNK, PKR, and IKK), each linked to metabolic disorders [38]:

(1) Obesity and Insulin Resistance:

ERS activates c-Jun N-terminal kinase (JNK), which phosphorylates IRS1 at serine residues, impairing insulin receptor signaling and inducing insulin resistance [39,40,41]. Global JNK deficiency prevents weight gain, whereas JNK activation promotes obesity [42].

(2) Lipid Metabolism Dysfunction:

ERS disrupts apolipoprotein B100 (ApoB100) folding, reducing VLDL assembly/secretion and causing hepatic triglyceride (TG) accumulation [43]. These metabolic disturbances—severe obesity, insulin resistance, and dyslipidemia—worsen NAFLD and ALD progression.

#### 3.1.3. ROS-Mediated Lysosomal Dysfunction

ROS exert dual opposing effects on lysosomal function that collectively impair cellular homeostasis: By inducing lipid peroxidation of lysosomal membranes, ROS increase membrane permeability, causing leakage of cathepsins into the cytosol, where they promote proteolysis, while hydroxyl radicals (OH) generate protein carbonyl derivatives through side-chain oxidation, leading to structural alterations, cross-linking, and enhanced proteasomal degradation [37].

Paradoxically, ROS simultaneously suppress autophagic flux by oxidizing critical amino acid residues in lysosomal enzymes to inhibit their catalytic activity [44], while also disrupting microtubule stability and soluble N-ethylmaleimide-sensitive factor (SNARE) protein function vesicle-associated membrane protein 8 (VAMP8)/syntaxin 17 (STX17) to impair autophagosome–lysosome fusion [45]. This autophagic blockade exacerbates hepatic steatosis by preventing lipid droplet clearance [46,47] and triggers Nod-like receptor pyrin-containing protein 3 (NLRP3) inflammasome activation through accumulated damaged organelles/proteins, amplifying IL-1β/IL-18 release [48,49]. Furthermore, oxidative damage to lysosomal membranes activates HSCs, directly promoting fibrogenesis [37,50].

#### 3.1.4. ROS-Mediated Activation of Inflammatory Signaling Pathways

Beyond ER stress and lysosome-mediated autophagy dysfunction, ROS activate inflammatory pathways through multiple mechanisms. As a core component of inflammatory responses, the NLRP3 inflammasome is directly activated by ROS through either oxidation of NLRP3 protein or mitochondrial damage-induced mtDNA release. Activated NLRP3 complexes with ASC (apoptosis-associated speck-like protein) and pro-caspase-1 to form the inflammasome, which cleaves pro-IL-1β and pro-IL-18 into their mature forms, triggering inflammatory cell recruitment and amplifying inflammatory cascades [49].

ROS further activate the MAPK pathway by stimulating upstream kinases (ASK1 and MEKK1), leading to ERK activation and nuclear translocation of transcription factors (AP-1 and ATF2) that upregulate pro-inflammatory cytokines (TNF-α and IL-6) and fibrogenic genes (TGF-β) [51,52]. In IL-6 signaling, ROS promote receptor dimerization and JAK kinase activation, resulting in STAT protein phosphorylation. Phosphorylated STAT dimers then translocate to the nucleus to induce expression of IL-6 and anti-apoptotic genes [53].

ROS-induced cytokines (TNF-α and IL-1β) reduce mitochondrial membrane potential and ATP production, further increasing ROS leakage. This creates a vicious cycle where elevated ROS activate NF-κB and JNK pathways to amplify cytokine production [54,55]. Additionally, ROS-stimulated fibrogenic factors (TGF-β and PDGF) activate quiescent HSCs, transforming them into collagen-producing myofibroblasts. Activated HSCs excessively synthesize extracellular matrix (ECM) components while suppressing matrix metalloproteinase (MMP) activity, collectively exacerbating fibrotic progression [55].

As central regulators, ROS drive hepatic fibrosis through multifaceted inflammatory activation—directly modifying inflammasome components, amplifying cytokine signaling via MAPK/JAK-STAT pathways, and sustaining a self-perpetuating oxidative stress-inflammation cycle that ultimately activates HSCs and disrupts ECM homeostasis.

#### 3.1.5. ROS-Mediated Dysregulation of Lipid Metabolism

ROS induce mitochondrial dysfunction, impairing hepatic β-oxidation of FFAs and leading to lipid metabolism dysregulation, resulting in hepatic steatosis. Both alcohol-induced and metabolic disorder-associated mitochondrial dysfunction exacerbate FFA accumulation, establishing a vicious cycle of lipotoxicity and oxidative stress.

Beyond indirect effects through mitochondrial impairment and inflammatory pathway activation, ROS directly modulate lipid metabolism through multiple mechanisms. In hepatocytes, AMP-activated protein kinase (AMPK) plays a crucial role in maintaining mitochondrial function and promoting autophagy. AMPK activation enhances fatty acid oxidation and reduces lipid accumulation; however, ROS can directly oxidize the α subunit of AMPK, inhibiting fatty acid oxidation while promoting lipogenesis and hepatic steatosis [56].

Sterol regulatory element-binding protein 1c (SREBP-1c), a master transcriptional regulator of hepatic lipogenesis, shows markedly elevated expression (5-fold higher than controls) in steatotic livers [12]. ROS activate SREBP-1c, which upregulates downstream targets including acetyl-CoA carboxylase (ACC) and FFAs, thereby promoting fatty acid and triglyceride synthesis. Additionally, ROS oxidatively modify the DNA-binding domain of peroxisome proliferator-activated receptor α (PPARα), a key regulator of fatty acid oxidation, suppressing its transcriptional activity and contributing to lipid accumulation [57].

Concurrent with these metabolic disturbances, hepatic fibrogenesis progresses through ROS-mediated mechanisms. Hepatocyte inflammation and altered immune microenvironments prompt Kupffer cells to release ROS and TGF-β, activating HSCs. Activated HSCs significantly increase collagen production, ultimately driving liver fibrosis [58].

#### 3.1.6. Additional Mechanisms of ROS Action in Hepatocytes

Beyond mitochondrial DNA damage, ROS directly target nuclear DNA and RNA through multiple oxidative pathways. The nucleobase guanine (G), possessing the lowest oxidation potential, is particularly vulnerable to ROS attack. Hydroxyl radicals (OH) specifically oxidize the C8 position of guanine, forming the mutagenic adduct 8-hydroxy-2′-deoxyguanosine (8-OHdG)—a well-characterized biomarker of oxidative DNA damage [59,60]. Peroxynitrite interacts with both DNA bases and sugar moieties, inducing DNA strand breaks. Due to its low oxidation potential, the most vulnerable DNA base attacked by ROS/RNS is guanine, leading to the formation of 8-nitroguanine [8]. More severely, ·OH can penetrate cellular and nuclear membranes to directly attack DNA backbone structures, inducing both single- and double-strand breaks (DSBs), with DSBs representing the most catastrophic form of genomic damage [61].

ROS also drive extensive lipid peroxidation cascades, oxidizing polyunsaturated fatty acids in cellular membranes and cytoplasm to generate highly reactive aldehyde byproducts, including 4-HNE, 4-hydroperoxy-2-nonenal (HPNE), and MDA. These electrophilic intermediates readily form covalent adducts with cellular proteins and nucleic acids [62,63], establishing a self-perpetuating cycle of macromolecular damage that exacerbates hepatocyte dysfunction and death. ONOO− can cause severe damage to amino acids and proteins by modifying them through the oxidation or nitration of tyrosine, tryptophan, methionine, and other amino acid residues [64].

The core mechanisms of ROS in steatotic liver disease are as follows: (1) mitochondrial dysfunction, (2) endoplasmic reticulum stress, (3) lysosomal impairment, (4) inflammatory pathway activation, (5) lipid metabolism dysregulation, and (6) DNA damage. Key cellular components and molecules are labeled to illustrate the multi-pathway hepatotoxic effects of ROS.

### 3.2. Differential Mechanisms of ROS in ALD Versus NAFLD

#### 3.2.1. The Role of ROS in ALD

There are significant epidemiological differences in ALD between Eastern and Western populations. Western populations demonstrate substantially higher rates of alcohol-induced cirrhosis and hepatocellular carcinoma, which correlates with their greater prevalence of heavy drinking and higher per capita alcohol consumption. In contrast, the incidence of alcoholic steatohepatitis has increased rapidly in recent years, particularly among young adults aged 19–44 years and women. Studies reveal that while Western populations require daily alcohol intake exceeding 80 g to significantly increase cirrhosis risk, Eastern populations show elevated risk at just 60 g/day, with women being particularly susceptible—cirrhosis may develop with as little as 20 g/day [2]. These differences suggest that beyond drinking patterns, variations in alcohol metabolism may play a key role. Eastern populations have a higher frequency of the ALDH2×2 gene mutation, which impairs acetaldehyde metabolism, along with greater CYP2E1 activity that promotes ROS generation during alcohol metabolism [65].

ALD also exhibits marked gender differences, with women being more vulnerable to alcohol’s toxic effects and more prone to developing ALD. This heightened susceptibility may stem from women’s lower alcohol dehydrogenase (ADH) activity, which shifts alcohol metabolism toward the microsomal ethanol-oxidizing system (MEOS) pathway [66]. The increased sensitivity of Eastern populations and women to alcohol-induced liver damage strongly implicates ROS as a critical mediator in ALD pathogenesis. ROS-induced oxidative stress appears to play a pivotal role in the initiation and progression of ALD. Alcohol consumption induces rapid elevation of ROS levels, while chronic alcohol exposure leads to persistent ROS stimulation combined with the synergistic effects of ethanol and its metabolites, resulting in more acute and severe oxidative damage.

The combined action of alcohol and ROS potently inhibits multiple hepatic enzymes, significantly disrupting lipid metabolism through dual inhibition of AMPK, a critical regulator of fatty acid β-oxidation. Notably, SREBP1c expression is rapidly upregulated by acetaldehyde and ROS following acute alcohol exposure, with this induction occurring more rapidly than the gradual stimulation observed in NAFLD, leading to pronounced hepatic lipid accumulation [67,68]. Epidemiological studies confirm this accelerated pathogenesis, with hepatic steatosis developing within two weeks of heavy drinking and present in 90% of moderate-to-heavy drinkers [26].

Alcohol-derived aldehydes and ROS act synergistically to promote extensive lipid peroxidation. Liver biopsy specimens from chronic drinkers demonstrate the formation of carcinogenic exocyclic DNA adducts through 4-HNE-DNA reactions, which impair DNA function while accelerating fibrotic progression [69,70]. Concurrently, MDA accumulation induces cross-linking of cellular macromolecules, including proteins and nucleic acids. These reactive aldehydes and ROS collectively activate HSCs, driving accelerated fibrogenesis and cirrhosis development [71]. Consequently, chronic alcohol exposure induces hepatic fibrosis more rapidly than NAFLD, with 10–20% of ALD patients developing fibrosis—a significantly higher rate than observed in NAFLD—reflecting the more aggressive oxidative stress and tissue damage in ALD [27].

An intriguing clinical observation is that while ALD progression is rapid during active drinking, abstinence can effectively halt disease advancement. Hepatic steatosis and inflammation typically show significant improvement within weeks to months of cessation, with some patients demonstrating fibrosis regression [72,73]. This phenomenon may relate to unique adaptive mechanisms in alcohol-exposed livers. In vitro studies using ethanol-treated hepatocytes and HepG2 cells reveal that chronic alcohol exposure not only induces CYP2E1 and ROS production but also upregulates glutathione (GSH), glutathione-S-transferase (GST), and heme oxygenase-1 (HO-1) through increased γ-glutamylcysteine synthetase expression, collectively mitigating oxidative damage [74]. Parallel research demonstrates alcohol-induced activation of Nrf2 and subsequent upregulation of CYP2A5, which contributes to ROS clearance [75]. These adaptive responses persist after alcohol withdrawal, enabling continued ROS neutralization and oxidative stress resolution.

#### 3.2.2. The Role of ROS in NAFLD

A strong positive correlation exists between the degree of oxidative stress/ROS elevation and disease severity in NAFLD patients. Longitudinal studies have demonstrated a progressive and significant increase in both serum and hepatic ROS levels during disease progression from simple steatosis to NASH and subsequent fibrosis [76]. Comparative analyses reveal that NASH patients exhibit substantially elevated serum ROS concentrations compared to those with simple steatosis, while hepatic ROS levels show a stage-dependent increase that strongly correlates with fibrosis severity (F2-F4 vs. F0-F1 stages) [65].

The oxidative stress biomarkers malondialdehyde (MDA) and 8-hydroxy-2′-deoxyguanosine (8-OHdG) serve as direct molecular evidence of ROS-induced oxidative damage. Their concentrations demonstrate significant positive correlations with histological grading of steatosis, lobular inflammation, and fibrosis staging. Clinical data indicate that serum MDA levels are significantly elevated in NAFLD patients compared to healthy controls, with NASH patients showing further increases [77]. Similarly, quantitative immunohistochemical analyses reveal that hepatic 8-OHdG levels exhibit a strong positive correlation with the degree of liver fibrosis [77,78].

The development of antibodies targeting nitrotyrosine has significantly advanced research on peroxynitrite. Numerous animal and large-scale clinical studies have detected nitrotyrosine in the tissues of obese and diabetic patients, suggesting its potential role in the diagnosis of NAFLD [79]. More recently, studies have proposed that molecular fluorescent probes for real-time detection of ONOO− levels exhibit higher sensitivity and efficacy than tissue staining or serum biomarker assays in assessing early-stage toxicity and diagnosis of NAFLD [80].

The pathophysiology of ROS accumulation in NAFLD involves both increased production and impaired clearance mechanisms. A critical determinant of ROS homeostasis is the cellular antioxidant defense system, particularly the enzymatic antioxidant capacity. Comparative studies demonstrate significant reductions in the activities of key antioxidant enzymes—including superoxide dismutase (SOD) and glutathione peroxidase (GPx)—in NAFLD patients relative to healthy controls, with the most pronounced deficiencies observed in NASH patients [81]. Importantly, patients with compromised antioxidant defenses show a three-fold higher incidence of liver fibrosis (45% vs. 15%), suggesting that antioxidant depletion may represent a critical driver of disease progression from steatosis to NASH and fibrosis. These findings are supported by multiple clinical studies that consistently demonstrate significant associations between ROS levels, oxidative damage markers, antioxidant capacity, and NAFLD progression [82,83], providing compelling evidence for the central role of oxidative stress in NAFLD pathogenesis.

The development of NAFLD is primarily driven by insulin resistance and dysregulated lipid metabolism, with accumulated ROS subsequently inducing organelle and cellular damage through oxidative stress—representing the “second hit” in NAFLD pathogenesis.

The metabolic disturbances caused by ROS in NAFLD differ significantly from those in ALD. ROS-mediated mechanisms in NAFLD include the following:

(1) Activation of inflammatory cytokines that interfere with insulin receptor substrate (IRS) phosphorylation, exacerbating insulin resistance [84,85].
(2) Inhibition of fatty acid oxidation through various inflammatory signaling pathways, promoting lipid accumulation and worsening hepatic steatosis [86].

These parallel processes synergistically accelerate NAFLD progression.

During early NAFLD stages, elevated FFAs stimulate mitochondrial activity, generating excessive ROS that damage mitochondrial structure and function. As disease progresses, accumulated mitochondrial dysfunction suppresses respiratory chain activity, creating an energy imbalance that impairs ROS clearance. This vicious cycle worsens hepatic lipid accumulation and oxidative stress, ultimately promoting hepatocyte necrosis/apoptosis and driving NASH and fibrogenesis [87,88].

NAFLD severity correlates strongly with the degree and duration of metabolic dysfunction: 70% of type 2 diabetes patients develop NAFLD; 90% of NAFLD patients are overweight; and 70% exhibit morbid obesity [89].

Unlike ALD, NAFLD features gradual but persistent ROS production that is difficult to resolve without addressing the underlying metabolic abnormalities. This results in insidious disease onset, with established NASH and fibrosis proving particularly refractory to treatment and carrying a poor prognosis.

### 3.3. NAFLD with Concurrent ALD (MetALD)

**Figure 4:**
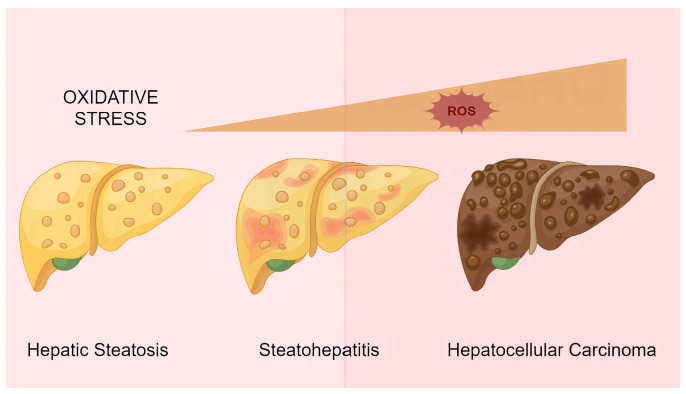
ROS-induced liver damage. Continuous oxidative stress can transform the liver through simple steatohepatitis, steatohepatitis, and even hepatocellular carcinoma.

As our understanding of NAFLD and ALD deepens, particularly regarding their shared pathogenic mechanisms, it has become evident that sustained oxidative stress damage driven by both conditions can lead to irreversible histological changes (Figure 4). The latest guidelines on fatty liver disease definitions have introduced the term *MetALD* (metabolic dysfunction and alcohol-associated liver disease) to describe the overlapping state between NAFLD and ALD [90]. Epidemiological studies demonstrate that MetALD exhibits a prevalence of 5.8% and a unique clinical phenotype (65% of cases), characterized by a greater metabolic disease burden (88% obesity and 62% diabetes/insulin resistance) and more severe hepatic injury (32% elevated ALT/AST, 18.5% FIB-4 ≥ 1.3) compared to NAFLD alone [86]. Although the current MetALD diagnostic framework excludes heavy drinkers (>60 g/day) and does not fully capture metabolism–alcohol interactions, it provides clinically relevant insights by identifying targetable oxidative stress pathways (Nrf2/FXR) and supporting combined therapeutic strategies (alcohol abstinence with metabolic optimization). This classification represents an advancement in precision hepatology, though further research is needed in biomarker development, targeted clinical trials, and mechanistic exploration to optimize its clinical utility [91].

## 4. Prevention and Therapeutic Approaches for ALD and NAFLD

The prevention and treatment of ALD and NAFLD share common features while exhibiting distinct differences, primarily stemming from their divergent sources and mechanisms of ROS generation and action. Fundamentally, antioxidant therapies for both conditions focus on two key aspects: reduction in ROS production and alleviation of oxidative stress damage. Lifestyle modification represents a crucial preventive measure applicable to both diseases.

Current therapeutic strategies under investigation for both ALD and NAFLD include several pharmacological approaches: conventional antioxidants (e.g., vitamin E and N-acetylcysteine); mitochondria-targeted antioxidants (e.g., MitoQ); ER stress modulators (e.g., 4-PBA); and enzyme inhibitors targeting ROS-generating systems (e.g., NOX inhibitors). These interventions either directly suppress ROS-generating pathways or enhance ROS scavenging capacity to mitigate oxidative injury.

### 4.1. Prevention of ALD and NAFLD

Strict lifestyle management plays an equally important role in both ALD and NAFLD, with many similarities between the two conditions. Dietary modifications, regular exercise, weight management, and smoking cessation have all been proven to effectively ameliorate oxidative stress in patients with either ALD or NAFLD [92].

#### 4.1.1. Abstinence from Alcohol

Alcohol abstinence constitutes the cornerstone intervention for ALD prevention and treatment, while it is equally critical for NAFLD management. Clinical studies demonstrate that six months of abstinence significantly reduces serum oxidative stress markers (MDA and 8-OHdG) and improves hepatic function parameters in ALD patients [93]. Longitudinal follow-up studies reveal substantially improved 5-year survival rates among abstinent ALD patients compared to continued drinkers [94,95]. Notably, even moderate alcohol consumption exacerbates hepatic steatosis and increases fibrosis risk in NAFLD patients, warranting complete alcohol avoidance in this population [96].

#### 4.1.2. Comprehensive Lifestyle Management

(1) **Dietary Intervention**

Adoption of a Mediterranean diet rich in antioxidants, monounsaturated fatty acids, and dietary fiber, while restricting refined carbohydrate intake, has been demonstrated to ameliorate hepatic steatosis and oxidative stress in NAFLD patients [97,98].

(2) **Exercise Regimen**

Moderate-intensity aerobic exercise (150 min/week) enhances antioxidant enzyme activity in NAFLD patients. Animal studies confirm that exercise attenuates alcohol-induced liver injury through activation of the Nrf2 pathway [94,99].

(3) **Weight Control**

Weight reduction of 5–10% represents an established therapeutic strategy for fatty liver disease, significantly improving hepatic steatosis and oxidative stress in NAFLD patients while ameliorating insulin resistance (IR) and reversing NASH histopathology [100,101]. Notably, obesity exacerbates alcohol-induced oxidative stress, rendering weight management equally crucial for ALD patients [102].

#### 4.1.3. Tobacco Control

Tobacco smoke contains abundant free radicals and pro-oxidants, serving as a significant exogenous source of oxidative stress that directly elevates hepatic ROS levels. Smoking cessation therefore represents a crucial intervention for ameliorating oxidative stress in both ALD and NAFLD patients. A large-scale cohort study found that the progression of NAFLD in smokers of all ages was significantly higher than that in non-smokers and was significantly related to the duration and amount of smoking [103]. In addition, smoking can also aggravate alcohol-induced liver injury, and smoking cessation can improve liver function indexes in ALD patients [104].

### 4.2. Therapeutic Approaches for ALD and NAFLD

The treatment of ALD and NAFLD currently represents both a challenge and a focus in research. As a critical mediator in both diseases’ progression, ROS has emerged as an important therapeutic target. Various antioxidant therapies have demonstrated promising efficacy in studies. However, while monotherapy with antioxidants has shown some benefits in preclinical studies or specific patient subgroups, the clinical results remain limited. With advancing understanding of disease mechanisms and application of novel technologies—including the updated concept of managing multi-morbidity and comorbidities, along with emerging targeted therapies and nano/molecular therapies—new opportunities are emerging for antioxidant treatment of ALD and NAFLD.

#### 4.2.1. Antioxidant Therapy

**Vitamin E and its derivatives**

Clinical studies demonstrate that vitamin E and its more potent isoforms (α-tocopherol and tocotrienols) significantly improve hepatic inflammation and fibrosis in NAFLD patients [105,106]. The natural antioxidant epigallocatechin gallate (EGCG) shows potential in animal and cellular studies by activating Nrf2 and AMPK pathways, with clinical evidence supporting its preventive and therapeutic benefits in NAFLD [107,108,109]. However, natural antioxidants have shown disappointing outcomes in ALD treatment, though vitamin E supplementation may still benefit ALD patients with nutritional deficiencies [102].

**Pentoxifylline (PTX)**

This methylxanthine derivative alleviates NASH progression by reducing ROS-induced lipid peroxidation and demonstrates therapeutic effects in both ALD and NAFLD [110,111].

**S-Adenosylmethionine (SAM) and Betaine**

SAM, a key methyl donor in liver disease, reduces oxidative stress by promoting glutathione synthesis. ALD patients with SAM deficiency show improved outcomes after supplementation (mortality reduction: 12% vs. 29%) [112,113,114]. Betaine enhances SAM levels via homocysteine remethylation, showing hepatoprotective effects in animal models of alcoholic steatosis [115,116,117,118]. Its phase II trial (NCT03073343) is evaluating ALT reduction in NAFLD [117].

**Controversies in Antioxidant Therapy**

A systematic review of 77 clinical trials found low-quality evidence supporting antioxidant efficacy in NAFLD, highlighting the need for well-designed RCTs [119]. While NOX1/4 inhibitors (e.g., GKT137831) reduce hepatic fibrosis in murine models [120,121], the pan-caspase inhibitor emricasan failed to improve outcomes in NASH-related cirrhosis in large trials [122,123].

**Therapeutic Limitations**

Although antioxidant enzyme activators may mitigate alcohol-induced oxidative stress in ALD, their efficacy in NAFLD remains limited since oxidative stress is secondary to core metabolic dysfunction. Nrf2 activators and similar targeted approaches show partial potential but require further validation [112,123].

#### 4.2.2. Targeted Therapy

The treatment of NAFLD and ALD is transitioning from traditional antioxidant support to precise targeted interventions. Although conventional antioxidants such as vitamin E demonstrate favorable safety profiles, their therapeutic effects remain limited to modest biochemical improvements without achieving histological reversal.

In March 2024, the FDA approved resmetirom (Rezdiffra™), the first drug specifically indicated for NASH with significant fibrosis (F2–F3), marking a watershed moment in hepatology [124]. Prior to this, no pharmacological treatments for NAFLD/NASH had received FDA approval, leaving clinicians to rely on lifestyle modifications and off-label metabolic drugs. The MAESTRO-NASH trial, a rigorous global phase 3 study, demonstrated that resmetirom alleviates hepatic steatosis, inflammation, and fibrosis through thyroid hormone receptor-β (THR-β) agonism—a novel mechanism distinct from previously unsuccessful candidates. This breakthrough validates decades of research on NASH pathogenesis and establishes THR-β as a viable drug target [125].

However, limitations temper enthusiasm. The trial’s 52-week histological improvement endpoint is a surrogate marker, leaving hard outcomes (e.g., cirrhosis prevention and mortality) unconfirmed. Enrollment biases (e.g., exclusion of advanced cirrhosis and underrepresentation of ethnic minorities) may limit generalizability, while the 25% response rate underscores the need for predictive biomarkers [126]. Long-term safety—particularly thyroid/cardiovascular effects—remains under review [127,128].

In regeneration studies, the FXR agonist OCA exhibited 100-fold greater efficacy than endogenous ligands in treating NASH-related fibrosis, with robust clinical benefits and safety evidence for improving NASH fibrosis. Although not yet FDA-approved, these findings support OCA’s use for NASH-induced fibrosis, particularly in combination therapies [129].

Resmetirom’s approval ushers in a new era of transforming NASH from “untreatable” to “modifiable”. However, its true impact hinges on real-world validation and iterative innovation. The journey has only just begun. Current research limitations that require future resolution include the following: (1) precision medicine: stratifying responders through genomic/proteomic analysis; (2) combination therapy: pairing resmetirom with GLP-1 agonists or FXR modulators to enhance efficacy; and (3) disease modification: extending trials to early NAFLD and compensated cirrhosis (F4).

In addition to the FDA-approved resmetirom therapy, modern antioxidant therapies have evolved from simple free radical scavenging to multidimensional modulation, including:

(1) **Mitochondria-Specific Protection**: Novel mitochondria-targeted antioxidants (e.g., MitoQ and SkQ1) selectively accumulate in the mitochondrial matrix, neutralizing ROS leaked from the electron transport chain [130]. Anti-oxCIN4 improves NAFLD phenotypes in WD-fed mice through three primary mechanisms: A) enhancing mitochondrial function (fatty acid oxidation); B) stimulating the antioxidant defense system (enzymatic and non-enzymatic); and C) protecting against impaired autophagy. Collectively, these findings support the potential application of Anti-oxCIN4 in NAFLD prevention/therapy [131].
(2) **Redox Signaling Modulation**: Selective regulation of NOX isoforms (e.g., inhibiting NOX4 while preserving NOX2-mediated immune function) maintains host defense while mitigating oxidative stress. Formononetin (FMN), a flavonoid with diverse bioactivities including antioxidant and anti-inflammatory effects, targets NOX4-based NADPH oxidase hyperactivity, enhances NADP/NADPH levels, and thereby promotes ferroptosis in activated HSCs, alleviating liver fibrosis [132].
(3) **Endoplasmic Reticulum-Targeted Therapy**: 4-Acetylantroquinonol B (4-AAQB), a natural ubiquinone derivative extracted from *Antrodia cinnamomea* mycelia, significantly ameliorates ER stress and inflammation in NAFLD mouse models as well as in J774A.1 and RAW264.7 cells [133]. The lipid-lowering drug fenofibrate improves NAFLD in high-cholesterol diet-fed mice by suppressing ERN1 and XBP1 expression, reducing MAPK8 phosphorylation, and alleviating ER stress [134]. Additionally, the insulin sensitizer pioglitazone has been shown to inhibit hepatic ER stress and insulin resistance in diabetic mice [135].
(4) **Metal-Based Nanozyme Therapy**: Nanomaterials with enzyme-like activities, termed nanozymes, feature metal-active centers as their key components. These centers effectively mimic catalytic redox processes, enabling them to emulate the activities of enzymes such as SOD and CAT [136]. The nanoparticles demonstrate robust ROS-scavenging capabilities, eliminating O₂⁻, H₂O₂, and ·OH while indirectly suppressing ONOO− formation. Animal studies have shown promising therapeutic outcomes [137].

These innovative approaches demonstrate that precise targeting of key nodes in oxidative stress pathways significantly enhances therapeutic outcomes. Notably, with the advancement of emerging technologies such as targeted therapy and CRISPR-based gene-editing nanocarriers, it is anticipated that related drugs will soon enter clinical trials.

#### 4.2.3. Treatment of Comorbid Metabolic Disorders

Fatty liver disease is not merely hepatic steatosis but represents a complex systemic metabolic dysfunction, necessitating concurrent management of systemic metabolic abnormalities to control associated oxidative stress.

Foremost among recent breakthroughs is this year’s star drug class—GLP-1 receptor agonists—which induce clinically significant weight loss (≥5% in 60% of patients) while reducing hepatic fat content by 30–40% (MRI-PDFF data) [138].

SGLT2 inhibitors exhibit a unique “starvation-mimicking” mechanism that upregulates antioxidant enzyme expression while reducing oxidative stress markers [139]. The phase III EMPEROR trial [140] demonstrated that empagliflozin significantly decreases systemic oxidative stress markers (e.g., TNF-α receptor) through modulation of the circulating proteome, suppresses inflammatory pathways (IL-6/STAT3), and improves myocardial energy metabolism. This study confirmed that this drug class provides multi-target benefits beyond glycemic control, particularly for diabetic comorbidities.

The PPAR-α/γ dual agonist saroglitazar improves hepatic inflammation and insulin sensitivity. A phase II clinical trial showed saroglitazar significantly ameliorated ALT, liver fat content (LFC), insulin resistance, and atherogenic dyslipidemia in NAFLD/NASH patients [141] (ClinicalTrials.gov Identifier: NCT03061721).

However, critical challenges remain to be addressed, i.e., limited efficacy in advanced fibrosis (F4 improvement rate < 15%), insufficient long-term safety data for combination therapies (lack of >5-year follow-up), and imprecise patient stratification (current biomarker accuracy requires improvement) [142].

#### 4.2.4. Treatment of NAFLD with ALD Comorbidity

The MetALD concept has provided important insights into managing NAFLD-ALD overlap, yet it still fails to fully address the therapeutic needs of this patient population. Currently, there are no unified standards for managing NAFLD and ALD overlap.

As with any other disease, the cornerstone of MetALD treatment should address its underlying etiology [90]. For obese patients, first-line therapy includes physical activity and dietary interventions, while diabetic patients typically require glucose-lowering medications. In severe ALD cases, psychosocial interventions for alcohol cessation and pharmacotherapy for alcohol use disorder (AUD) are crucial. However, current treatment strategies have significant limitations—isolated metabolic interventions (e.g., weight or glycemic control) may not fully counteract alcohol-induced hepatotoxicity, while alcohol abstinence alone may be insufficient, as persistent metabolic dysfunction can still drive disease progression in MetALD patients. Consequently, many MetALD patients ultimately require additional therapies [91].

To date, pharmacotherapy for steatohepatitis has primarily focused on NAFLD. Nevertheless, given the shared pathogenic pathways between NAFLD and ALD, drugs developed for NAFLD may also hold promise for ALD and MetALD [143].

Emerging evidence suggests the following two drug classes, which may be particularly beneficial for MetALD:

**GLP-1 receptor agonists**—Beyond ameliorating steatohepatitis, they modulate the dopamine reward system, reducing alcohol cravings (animal models show a 50% reduction in alcohol consumption). Additionally, by delaying gastric emptying and enhancing satiety, semaglutide may indirectly mitigate alcohol-related harm [144,145]. A recent large retrospective study in obese patients found that semaglutide was associated with a 50–60% reduction in AUD incidence and relapse risk over 50 months compared to other anti-obesity medications, with similar findings in T2D cohorts [146]. Notably, due to its weight-loss effects, caution is warranted in patients with normal BMI [147].

**FGF21 analogs**—Efruxifermin and pegozafermin, two FGF21 analogs, have shown promising results in NASH resolution and fibrosis regression [148,149]. Given their ability to suppress CYP2E1 activity (thereby reducing oxidative stress from alcohol metabolism), they may also be highly relevant for MetALD. FGF21, a stress-induced hormone secreted by the liver and adipose tissue in response to high fructose or alcohol intake, has been shown to reduce alcohol consumption in animal models. While human data are lacking, FGF21 analogs—once approved for MASLD in coming years—are expected to gain attention for MetALD.

Drugs targeting lipid and/or bile acid metabolism (e.g., obeticholic acid and lanifibranor) may similarly benefit hepatic steatosis, inflammation, and even fibrosis, as dysregulated lipid metabolism triggers HSC activation. This mechanistic rationale may explain the anti-fibrotic effects observed in phase II/III trials [150,151].

Among investigational ALD therapies, larsucosterol (an endogenous oxysterol and epigenetic modulator inhibiting DNA methyltransferases) has shown encouraging results in phase I/IIa trials for alcohol-associated hepatitis, with phase IIb data anticipated soon [152].

In contrast, resmetirom may demonstrate limited efficacy in MetALD. Although thyroid hormone promotes hepatic lipid oxidation and improves insulin sensitivity, no significant dysfunction in this pathway has been reported in ALD. Thus, resmetirom’s benefits for MetALD may be modest, particularly in ALD-dominant cases.

#### 4.2.5. Therapeutic Distinctions Between ALD and NAFLD

Significant differences exist in antioxidant therapy approaches between ALD and NAFLD, primarily due to their divergent ROS generation and action mechanisms.

In ALD, the substantial ROS production from alcohol metabolism—combined with chronic alcohol consumption sustaining ROS generation—results in more severe and recalcitrant oxidative stress. This explains why alcohol abstinence remains the cornerstone of ALD management, as it directly limits ROS generation at its source. Consequently, antioxidant monotherapy has demonstrated limited efficacy in clinical ALD studies.

Conversely, NAFLD antioxidant strategies emphasize comorbid disease management (e.g., T2DM and obesity), which themselves exacerbate oxidative stress. NAFLD-associated ROS arises from multifactorial sources, and oxidative injury typically progresses insidiously. This chronic, low-grade ROS exposure may render antioxidant interventions more effective in NAFLD, as they have broader opportunities to modulate ROS production and clearance. Notably, emerging NAFLD therapies—including resmetirom, GLP-1 receptor agonists, and SGLT2 inhibitors—have demonstrated robust efficacy, partly through indirect antioxidant mechanisms.

## 5. Current Challenges and Future Perspectives

ROS play a pivotal role in the pathological progression of both ALD and NAFLD. Excessive ROS generation not only directly induces hepatocyte injury but also drives disease progression by activating inflammatory responses, exacerbating lipid metabolism dysregulation, promoting fibrosis, and triggering apoptosis. Therefore, a deeper understanding of the mechanisms underlying ROS-mediated damage in ALD and NAFLD is critical for developing effective therapeutic strategies.

With advancing technology, the integration of multi-omics signatures—including genomics, metabolomics, and microbiomics—into precision medicine, guided by artificial intelligence (AI) for optimized treatment regimens, presents novel opportunities not only for ALD/NAFLD therapy but also for other oxidative stress-related pathologies (e.g., ischemia–reperfusion injury). With advances in the integration of medicine and engineering, RNS fluorescent probe technology can now be utilized in clinical practice to evaluate the efficacy of therapeutic drugs. This progress foreshadows the emergence of more precise and sustained antioxidant treatment modalities.

Furthermore, improved management of comorbidities—such as incorporating diabetes management guidelines [11] into liver-related outcomes, establishing national NAFLD treatment quality metrics, and aligning real-world evidence with randomized controlled trial data—can enhance outcomes in fatty liver disease. Additionally, AI may help reduce the variability in histological assessments that currently challenges clinical trials. Finally, ongoing technological advancements will enable increasingly personalized approaches to managing NAFLD and ALD patients. These developments may lead to rapid evolution in current diagnostic and therapeutic recommendations for these conditions.

## Figures and Tables

**Table 1:** Mechanisms of ROS production in ALD and NAFLD.

		ALD	NAFLD
Similarities	Main sources of ROS Endoplasmic reticulum stress Inflammation simulation	Alcohol metabolism and FFA metabolism can lead to increased mitochondrial ETC electron leakage and ROS overproduction.	
		This results in an imbalance of calcium homeostasis and induces mitochondrial ROS production.	
		The activation of Kupffer cells and the release of inflammatory factors (such as TNF-α and IL-6) promote ROS production.	
Difference	Triggers	Alcohol metabolism is the main source of ROS production.	Insulin resistance and lipid metabolism disorders are the main drivers of ROS production.
	Generation pathways	CYP2E1 pathway: production of large amounts of O_2_^−^ and H_2_O_2_.	Insulin resistance and lipid metabolism disorders are the main drivers of ROS production.
		ADH/ALDH2 pathway: Alcohol metabolism produces NADH, which inhibits the mitochondrial respiratory chain and increases electron leakage.	Mitochondrial fatty acid beta oxidation: FFA overload leads to overload of the electron transport chain and increased electron leakage.
			ERS: FFA-induced Ca^2+^ leakage and mitochondrial ROS production.

## Abbreviations

The following abbreviations are used in this manuscript:

4-HNE	4-hydroxynonenal
4-PBA	4-phenylbutyric acid
8-OHdG	adduct 8-hydroxy-2′-deoxyguanosine
ADH	alcohol dehydrogenase
AKG	α-ketoglutarate
ALD	alcoholic fatty liver disease
ALDH2	aldehyde dehydrogenase 2
AMPK	AMP-activated protein kinase
ASH	alcohol-related hepatitis
ATF	activating transcription factor
DAG	diacylglycerols
ER	endoplasmic reticulum
ETC	electron transport chain
FFAs	free fatty acids
GSH	glutathione
GST	glutathione-S-transferase
HCC	hepatocellular carcinoma
HO-1	heme oxygenase-1
HSCs	hepatic stellate cell
IL-1β	interleukin
IRE1α	inositol-requiring enzyme 1α
MAPK	mitogen-activated protein kinase
MDA	malondialdehyde
MEOS	microsomal ethanol oxidizing system
mPTP	mitochondrial permeability transition pore
NAD+	nicotinamide adenine dinucleotide
NAFLD	nonalcoholic fatty liver disease
NASH	nonalcoholic steatohepatitis
NLRP3	Nod-like receptor pyrin-containing protein 3
NOX	NADPH oxidase
Nrf2	NF-E2 p45-related factor 2
PERK	PKR-like ER kinase
PINK1	PTEN-induced putative kinase 1
PPARα	peroxisome proliferator-activated receptor α
PTX	pentoxifylline
ROS	reactive oxygen species
SAM	S-adenosylmethionine
SERCA	sarcoplasmic/endoplasmic reticulum calcium ATPase
SOD/GPx	superoxide dismutase/glutathione peroxidase
SREBP-1c	sterol regulatory element-binding protein 1c
TBE-31	alkynyltricyclic dicyanenone
TCA	tricarboxylic acid cycle
TNF-α	tumor necrosis factor
TRAF2	TNF receptor-associated factor 2
TUDCA	taurine-conjugated ursodeoxycholic acid
UPR	unfolded protein response
VLDLs	very-low-density lipoproteins
XBP1s	X-box binding protein 1
